# A randomised cross-over trial of QT response to hyperventilation-induced anxiety and diaphragmatic breathing in patients with stress cardiomyopathy and in control patients

**DOI:** 10.1371/journal.pone.0265607

**Published:** 2022-03-23

**Authors:** George M. Watson, Jacalin Sutherland, Cameron Lacey, Paul G. Bridgman

**Affiliations:** 1 Department of Cardiology, Christchurch Hospital, Christchurch, New Zealand; 2 Department of Psychological Medicine, Christchurch Hospital, Christchurch, New Zealand; University of Dundee, UNITED KINGDOM

## Abstract

**Objectives:**

The most perfect example of the mind-body interaction in all of medicine is provided by stress cardiomyopathy. In stress cardiomyopathy, what is initially a purely emotional event may become rapidly fatal. Prolongation of the QT interval is a cardinal feature of the condition, but the mechanism of the prolongation is unknown. We undertook a randomised controlled trial of stress with a cross-over design, comparing the cardiac response of women with a history of stress cardiomyopathy to age-matched controls to explore the mind-body interaction. Our hypothesis is that the hearts of women with a history of stress cardiomyopathy will respond differently to emotional stress than those of the controls.

**Method:**

This is a randomised cross-over study. Each patient underwent two separate 24-hour Holter monitors performed at least 5 days apart. Baseline recording was followed by either the stress intervention (hyperventilation) or control (diaphragmatic breathing). Our primary endpoint is change in QTc interval over the first hour. Secondary endpoints were change in QTc over 24 hours, and change in SDNN, a measure of heart rate variability. As a secondary stressor, each participant was telephoned four times during their stressed recording and asked to complete a questionnaire.

**Results:**

Twelve stress cardiomyopathy patients and twelve control patients were recruited. Baseline characteristics did not differ between cases and controls. With hyperventilation, there was a significant initial difference in anxiety (p<0.001), heart rate response (p<0.0001), and QTc (p<0.0002) compared to diaphragmatic breathing, but no differences between the cases and controls. Only first phone call caused an increase in QTc in cases and controls (p = 0.0098). SDNN increased with hyperventilation (p<0.0001) but did not differ between cases and controls.

**Conclusions:**

QTc response in women with a history of stress cardiomyopathy does not differ from controls. The relevance of QT prolongation and sensitivity in the autonomic response to the pathogenesis of stress cardiomyopathy remains uncertain.

## Introduction

The mind-body interaction is of interest to the wider public and to physicians alike. The most perfect example in all of medicine is provided by stress cardiomyopathy. Stress cardiomyopathy can occur precipitously in an otherwise healthy person and may be rapidly fatal. In many cases, the clear-cut trigger for this physical condition is a purely emotional event. There are many case reports and case series of the condition. These observational studies are often of interest but have not directly explored the link between the emotion and the physical illness. Further research in the area would be helped by the development of a marker for the link.

Prolongation of the QT interval is a cardinal feature of stress cardiomyopathy [1]. Usually, the QT prolongation begins prior to presentation, and then progressively lengthens further, peaking typically on the third day of admission [2]. The mechanism of this prolongation is poorly understood. Its time course differs from that of the most common structural abnormalities: troponin rise and LV function changes [2].

QT prolongation has long been reported in response to emotional stress in healthy people and in those patients with the congenital long QT syndromes [3, 4]. In these syndromes, the mind-body link is also poorly understood. Further, the chain of causality is not known. Does an altered neurocardiac link predispose to stress cardiomyopathy, or does it come about as a result of it? For this reason, we look to examine the QT response to stress in patients who have had stress cardiomyopathy, comparing them to controls without significant cardiac pathology. In undertaking a randomised controlled trial of stress we seek to explore the mind-body interaction, looking for a marker of a difference between cases and controls. Our hypothesis is that the hearts of women with a history of stress cardiomyopathy will respond differently to emotional stress than those of age-matched controls.

## Methods

Approval for the study was obtained from the Health and Disability Commission’s Ethics Committee and the study was registered in the Australia and New Zealand Clinical Trials Registry (ACTRN12618001812280). The study was performed in an outpatient setting at Christchurch Hospital. Written informed consent was obtained from each participant.

Stress cardiomyopathy predominantly occurs in post-menopausal female patients [1]. To obtain a homogenous study population that reflects this demographic, we recruited post-menopausal women who were over the age of 55 years. Men were excluded from the study. All patients in the stress cardiomyopathy group had a history of the condition. Stress cardiomyopathy was defined using modified Mayo criteria [5]. Patients presented with a typical wall motion abnormality and a troponin rise without significant coronary artery disease. Echocardiographic resolution was documented at follow-up. For recruitment into the current study, we required a clear emotional stressor for the index episode. The control group was made up of patients who had been seen by the cardiology service for investigation of chest pain or palpitations but were free of significant cardiac pathology. All had documented normal echocardiograms. Exclusion criteria for the study were presence of an anxiety disorder, diabetes mellitus, atrial fibrillation, left bundle branch block, a significant coronary lesion, impaired LV function, or beta-blockade therapy.

The study is of randomised, controlled, cross-over design. Randomization was performed by GW for each study arm before enrolment began, using block randomisation with randomly sized blocks. Participants were enrolled by GW and PB and their details added to a spreadsheet in the order that they consented to the study. The sequence was implemented using a spreadsheet formula that revealed the allocation of the next participant once their details had been added. Once allocation was revealed, it was not hidden from either the participant or the investigators. Each woman attended two visits where, after a 24-hour Holter monitor was applied and a five-minute rested baseline recording was captured, a three-minute breathing exercise was carried out to either induce or relieve stress. Hyperventilation induces anxiety and so was used as the stressor [6]. Diaphragmatic breathing may relieve anxiety and so was used as the control [7]. Specific verbal instructions given to each patient are available in S2 Instructions to participants. The Holter monitors continued to record until 24 hours of data had been collected. Each woman was randomly assigned to either the hyperventilation-first or diaphragmatic breathing-first group, and then crossed-over after at least a 5-day washout period. As an additional stressor, each woman was also phoned four times during their stressed recordings to complete an emotional affect questionnaire. The calls were made without pre-warning the patient, with one call made to be close to their bedtime, another to be at the time they reported typically waking, and the two others made to give the evenest spread possible across the 24 hours.

The effect of each intervention on anxiety was assessed using a 5-point Likert scale immediately after the completion of the three-minute breathing exercise.

Our primary endpoint was the change in the QTc interval over the first hour.

Our secondary endpoints included QTc over the remainder of the 24-hour recording period and SDNN. SDNN is the standard deviation of the mean time difference between two R waves of normal heart beats calculated over a specified timeframe. SDNN was chosen as it is the most widely used method of assessing heart rate variability. It is calculated from analysis in the time domain and represents sympathetic nervous system activity at the heart [8]. We assessed change in SDNN over the first 20 minutes of the 24-hour recording period, measured in periods of 5 minutes.

After all patients had completed the study, each 24-hour Holter recording was analysed blind. The QT interval was measured at rest, at minute 4 of the baseline recording, and then during the breathing intervention, and at 2.5 minutes, 5 minutes, 7.5 minutes, 20 minutes, 60 minutes, 8 hours, 16 hours, and 24 hours after the end of the intervention. At each point, three measurements were made of the QT interval using the maximum slope intercept method [9]. These were corrected with Bazett’s formula and the mean of the three was recorded. At times where measurement of a QT interval at a certain timestamp was impossible due to movement artefact, the closest three intervals were measured. For points up until 20 minutes post-intervention, the acceptable window was less than 30 seconds before or after the target time. For the remaining time points, a deviation of five minutes either side was accepted. For the phone calls, three measurements were made 2 minutes before the phone call, and at 3, 8, and 13 minutes after. SDNN was calculated using the HScribe Holter monitor software (Welch Allyn, New York).

For the primary endpoint of QTc, a coefficient of variation of 5% was estimated. Using a cross-over study design, 24 patients were calculated to give the desired α = 0.05 and β = 0.80. Statistical analysis was performed using SAS 9.4. We used a multivariate repeated measures analysis of variance statistical design to assess the effect of the interventions over the 24-hour period, and the difference between the response of stress cardiomyopathy and control patients to each intervention. A doubly repeated measures analysis was used to compare the response to the hyperventilation intervention with the response to the diaphragmatic breathing intervention for the stress cardiomyopathy and control patients both separately and together. For baseline characteristics, p-values for continuous data were derived using the two-tailed student t-test for two independent groups with assumed heteroscedasticity, while p-values for categorical data were derived using Fisher’s exact test. To assess the anxiety level data from the Likert scale, a mixed-effects ordinal regression model was built. Anxiety score was set as the dependent variable, and the state (diaphragmatic breathing or hyperventilation) and group (case or control) were set as independent variables. The group status was also included as an interaction term. Statistical significance was taken as a 2-sided p<0.05.

Minimal changes were made to the protocol submitted to the clinical trial registry: the washout period was reduced from 7 days to 5 days and women without non-ST elevation acute coronary syndrome were accepted into the control group. The emotional affect questionnaire results were not analysed to reduce the risk of multiple comparisons yielding erroneously significant results.

## Results

**Fig 1:**
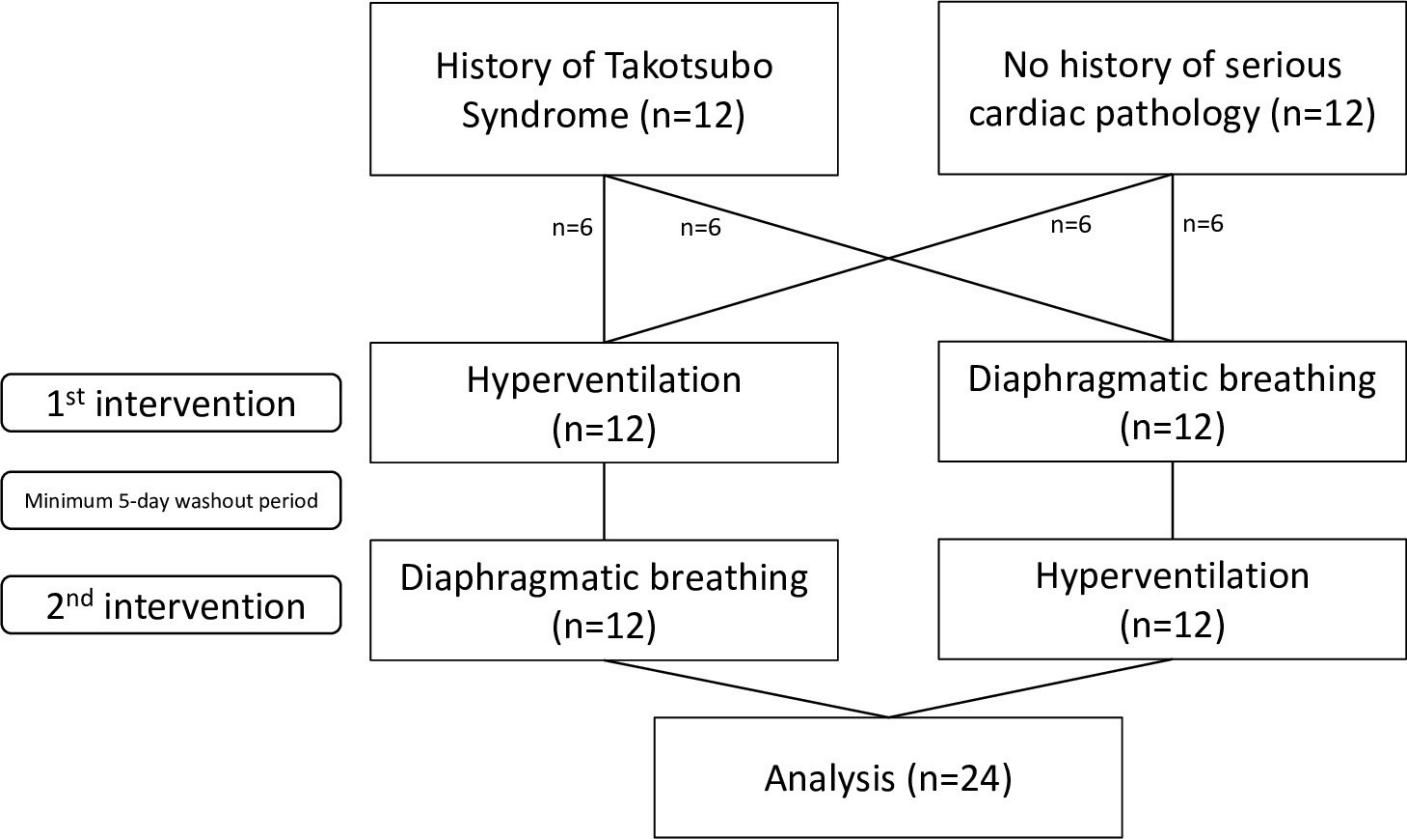
CONSORT flowchart showing number of patients recruited, assigned to each intervention, and analysed.

The study was performed between November 2018 and February 2019. Twelve stress cardiomyopathy patients and twelve control patients were enrolled and completed the study (Fig 1). Analysis was by originally assigned groups. Demographic details are presented in Table 1, showing that baseline characteristics did not differ between the cases and controls (all p = NS, see table for details). The index stress cardiomyopathy event in those patients occurred a mean of 2.1 years prior to the study (range 0.2 to 3.9 years).

**Table 1:** Baseline characteristics in cases and controls.

	Cases	Controls	
**Age (years)**	67.3 (SEM 2.9)	66.2 (SEM 2.5)	p = 0.79
**Systolic BP (mmHg)**	136 (SEM 4.2)	133 (SEM 3.5)	p = 0.59
**Diastolic BP (mmHg)**	82 (SEM 1.7)	77 (SEM 3.0)	p = 0.19
**BMI (kg/m²)**	25.1 (SEM 1.75)	26.3 (SEM 1.00)	p = 0.56
**History of hypertension**	7 (58%)	4 (33%)	p = 0.95
**Smoking pack year history**	7.42 (SEM 3.49)	8.58 (SEM 3.02)	p = 0.81
**Current smoker**	1 (8.3%)	0 (0.0%)	p = 1.00
**Weekly units of alcohol**	2.04 (SEM 0.76)	4.25 (SEM 1.10)	p = 0.13
**Ability to drive**	10 (83%)	11 (92%)	p = 0.50
**Living alone**	7 (58%)	5 (42%)	p = 0.89

**Fig 2:**
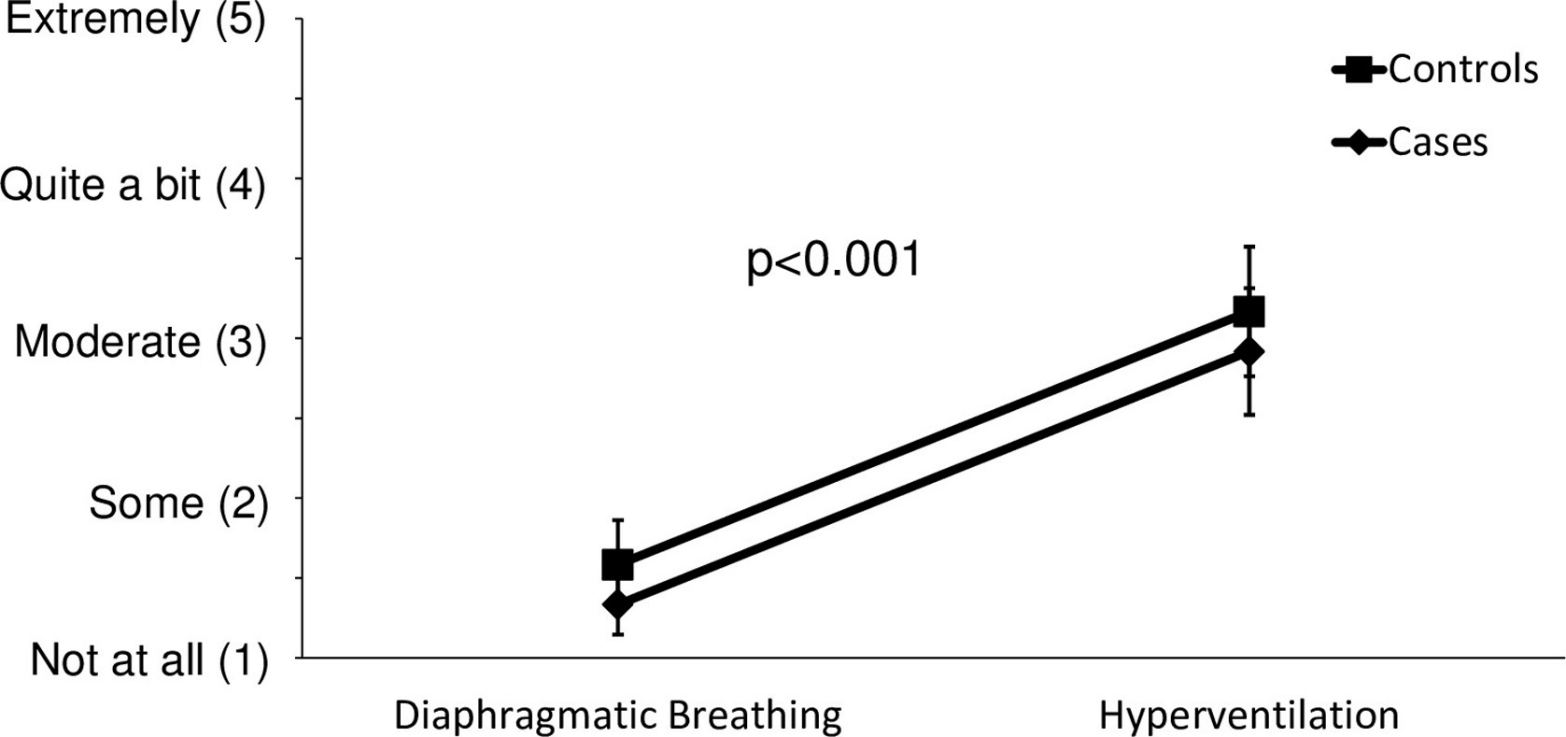
The effects of hyperventilation versus diaphragmatic breathing on subjective anxiety in cases and controls, as measured by a five-point Likert scale.

Hyperventilation resulted in a significant increase in anxiety levels in both groups (p<0.001), with no difference between the cases and controls, p = 0.939 (Fig 2).

**Fig 3:**
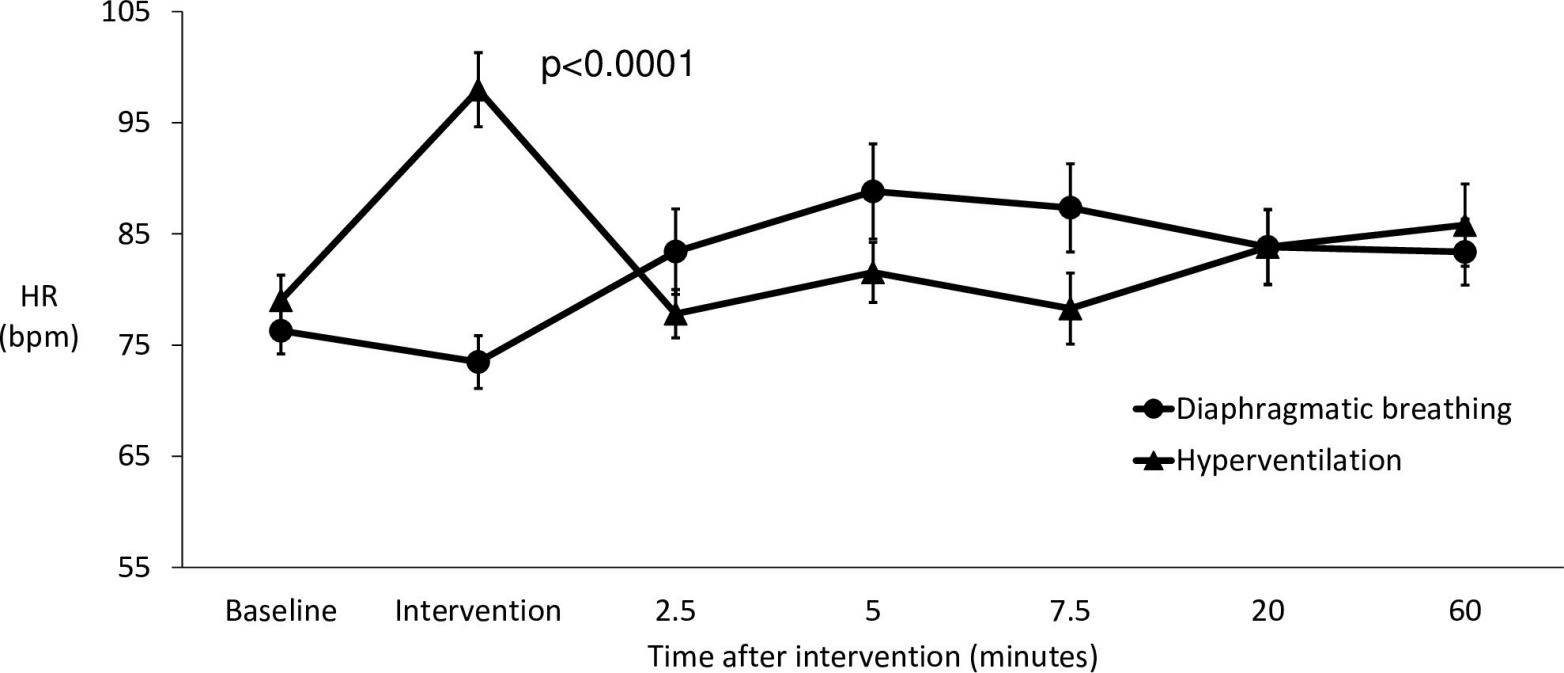
The effects of hyperventilation versus diaphragmatic breathing on the heart rate of all participants in the first 60 minutes.

During the breathing exercises, there was a highly significant initial difference in heart rate response between hyperventilation and diaphragmatic breathing (p<0.0001). However, within 2.5 minutes they were back at the same level, with no difference between them over the remainder of the first hour and the rest of the 24 hours (Fig 3).

**Fig 4:**
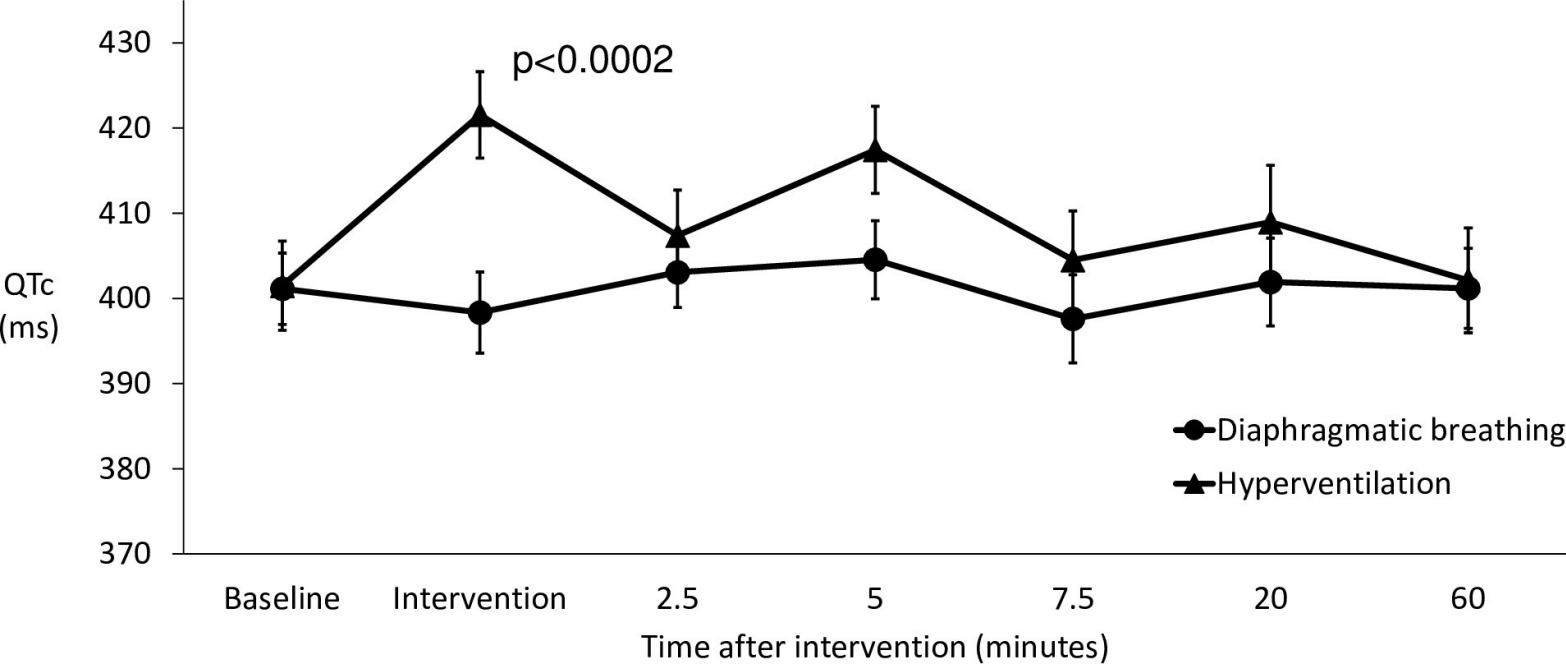
The effects of hyperventilation versus diaphragmatic breathing on the QTc of all participants in the first 60 minutes.

The QTc interval change induced by hyperventilation (p<0.0002) persisted longer than the heart rate increase. In the hyperventilation group QTc was lengthened for 20 minutes. At the one-hour mark it had returned to be equal with the diaphragmatic breathing group (Fig 4).

**Fig 5:**
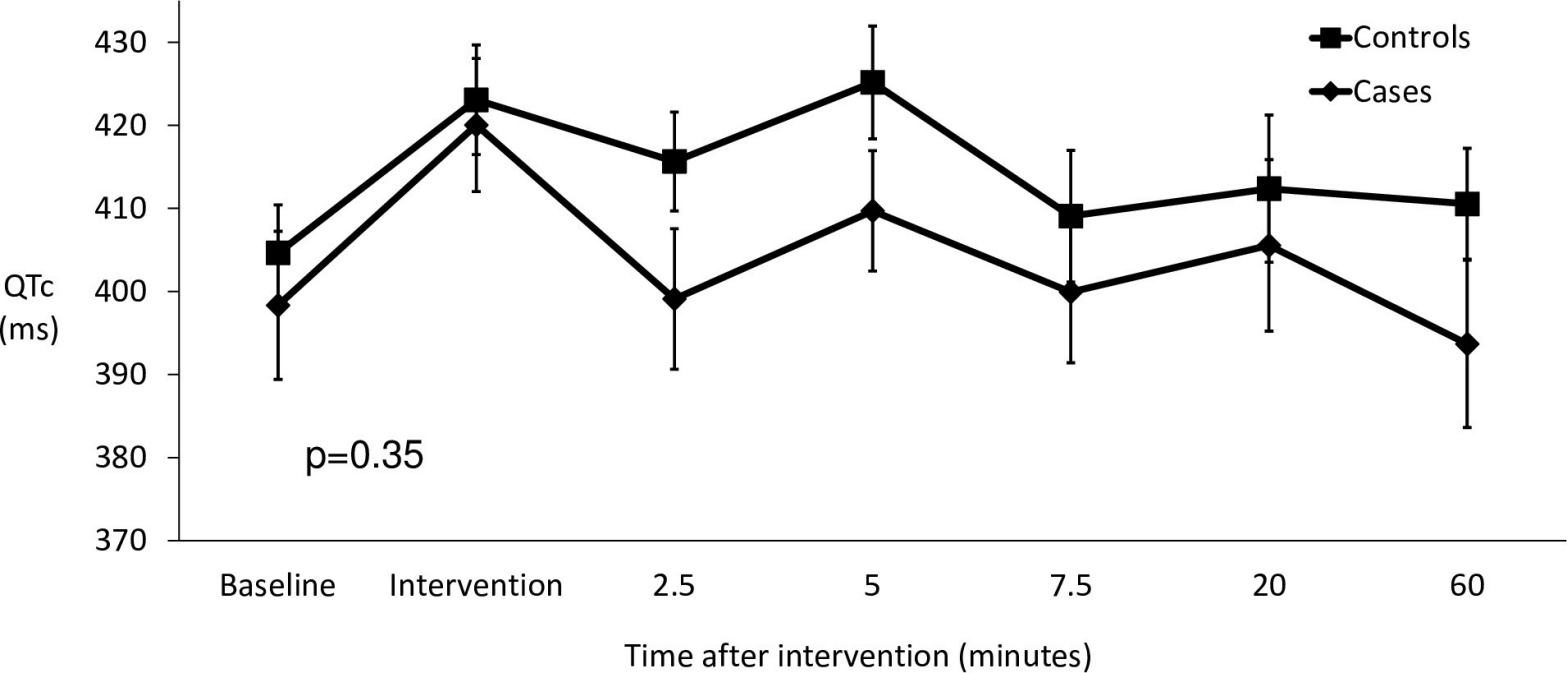
The effects of hyperventilation on the QTc of cases versus controls in the first 60 minutes.

There was no difference in QTc response between cases and controls (p = 0.35). With diaphragmatic breathing, QTc did not change. With hyperventilation anxiety, QTc increased to the same extent in both groups (Fig 5). No other interaction was found and there was no order effect (p = 0.91).

**Fig 6:**
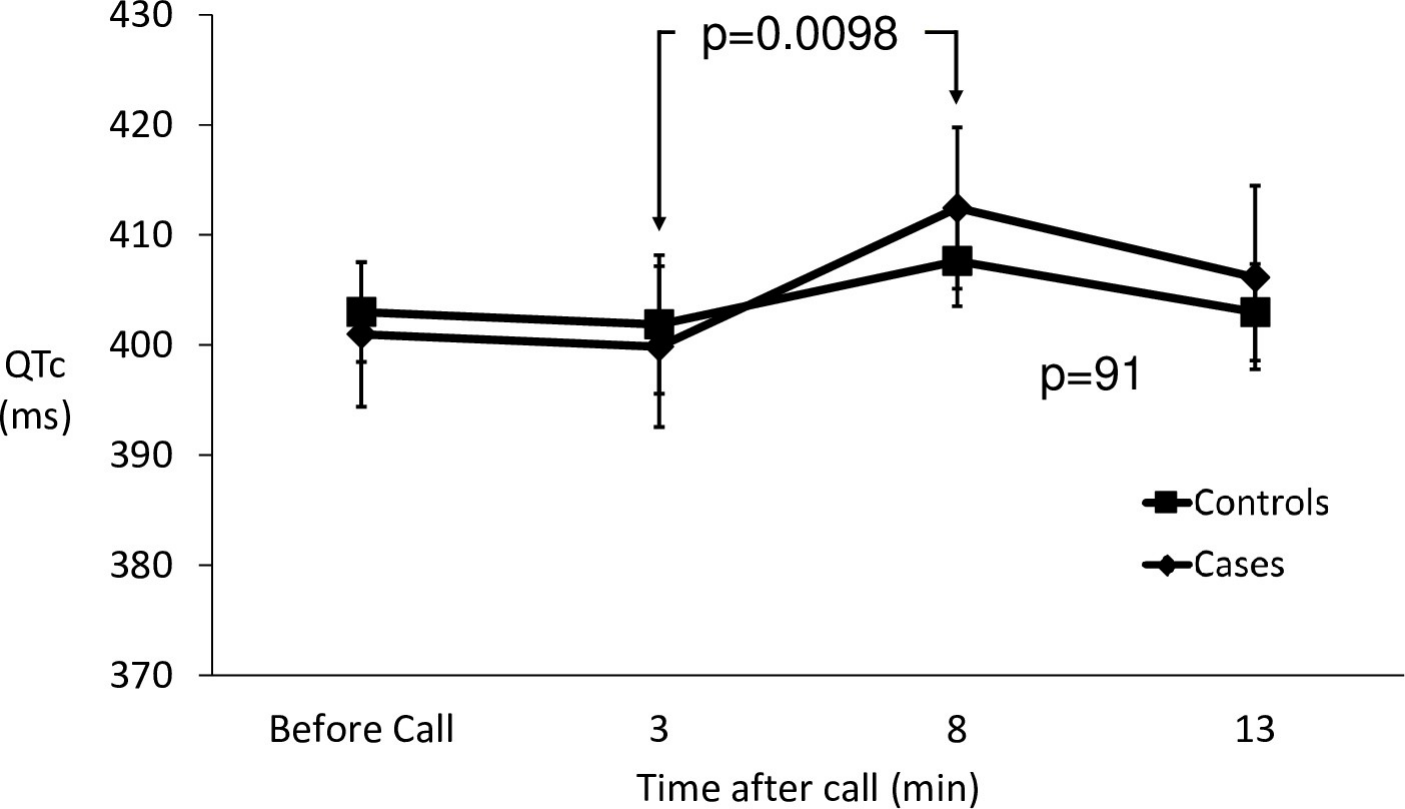
The effects of an unheralded phone call on QTc in cases and controls.

The first of the four phone calls caused no increase in heart rate but did result in a significant increase in the QTc in both groups (p = 0.0098) (Fig 6), with no difference between patients and controls (p = 0.91). It was noted by the investigator making the phone calls that whilst the first call did create anxiety in the women, the subsequent calls were something that they often looked forward to and there was no anxiety evident. With those subsequent calls there was no change in either heart rate or QTc.

**Fig 7:**
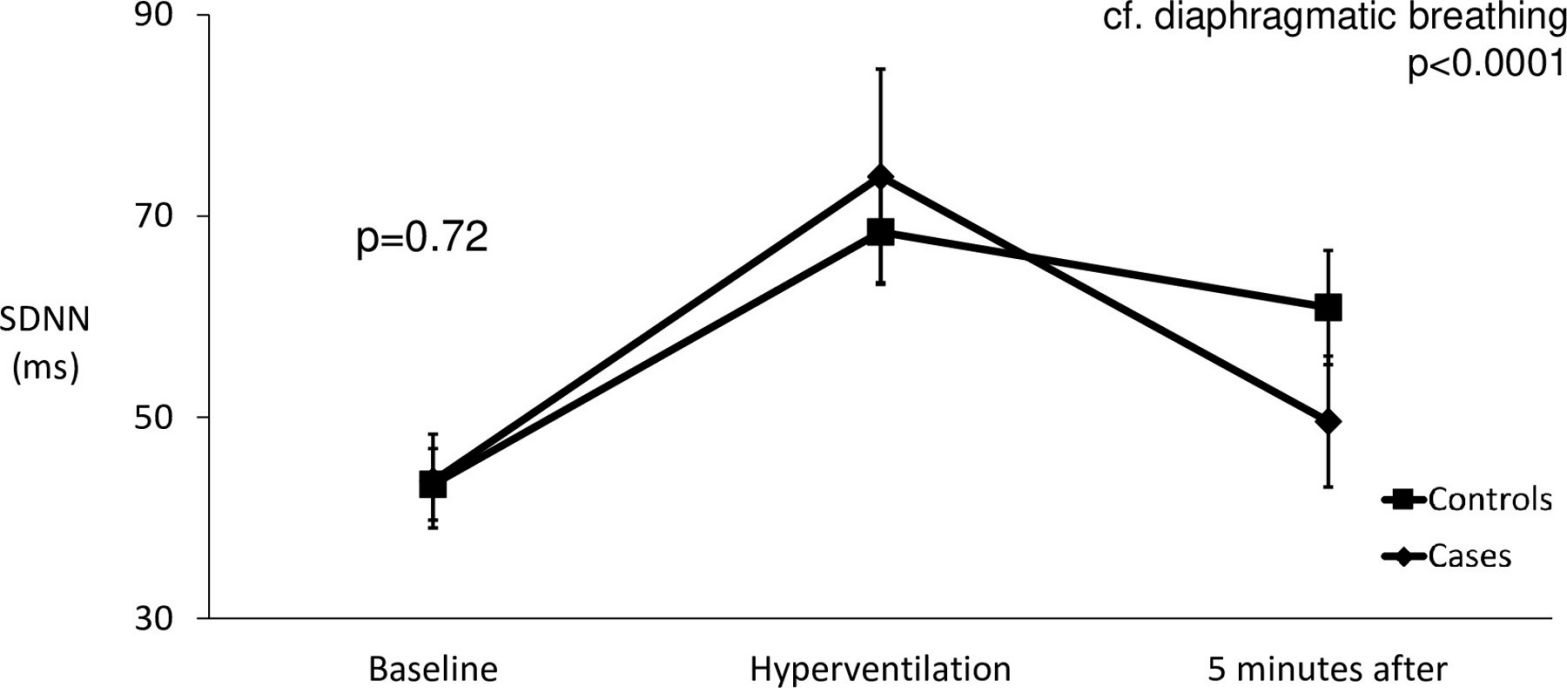
The effects of hyperventilation on SDNN in cases and controls.

Heart rate variability as assessed by SDNN increased in response to hyperventilation compared to diaphragmatic breathing (p<0.001) (Fig 7). There was no difference in the response of cases and controls (p = 0.72).

## Discussion

There is a dearth of prospective randomised controlled trials in stress cardiomyopathy. In this study of post-menopausal women, hyperventilation caused anxiety, increased heart rate, and prolonged QTc. Heart rate was increased during the hyperventilation but had returned to baseline by 2.5 minutes after. The QTc prolongation persisted for 20 minutes. An unheralded phone call was also effective in increasing the QTc. We found no difference in these QTc responses between the women with a history of stress cardiomyopathy and the women in the control group. This suggests that QTc interval change does not provide a marker of an altered neuro-cardiac link in these patients.

It has been long debated whether the cause for stress cardiomyopathy is in the brain, the heart, or even perhaps in the connection between the two. QT prolongation is a cardinal feature of the cardiomyopathy and, as our data has shown, is labile and strongly affected by emotion. However, there is nothing intrinsically different in response between the patients and the controls. We found no evidence that the cases have a permanent background alteration in QT sensitivity and also no evidence for them carrying forward any abnormality in QT response having had an episode. This makes it unlikely that variation in QT response will be a marker of risk in these patients. We therefore still do not have specific risk markers or a marker for the mind-body connection.

We specifically included only patients with stress cardiomyopathy where the index episode had an emotional trigger in an effort to select for women in whom the neuro-cardiac link might be most sensitive. However, a weakness of our study is that the hyperventilation only induced a moderate level of anxiety. Whilst this did provoke significant QTc prolongation, it is possible that had we subjected our cases to more intense emotional stress, for instance by misleadingly giving them very bad personal news, that we could have seen more of a response. However, we would not have been comfortable with the ethics of that as a research approach. In effect, this study took women with a history of stress cardiomyopathy and stressed them to see what happens. The ethical issue for future research along the lines of this study would be around how much stress is it reasonable to apply and informed consent.

It should also be noted that hyperventilation is not just a pure emotional stressor, it is a mixed stressor. It is well known to cause metabolic derangement, for instance increased pH and altered calcium binding [10]. We did not measure these in our patients and it is possible they could have been a factor in some of the heart rate changes that we observed. As a purely emotional stressor, we used the unheralded phone calls during the 24-hour monitoring period. Unlike during the hyperventilation, we did not measure anxiety with the Likert scale during these phone calls. However, we did observe that it was only the first call that was overtly stressful to the patients and that it was only the first call that resulted in a QTc response. This QTc response was smaller than that caused by the hyperventilation with moderate anxiety.

There is one published study with similarities to ours. In a Swedish study published in 2014, patients with a history of stress cardiomyopathy and controls were subjected to a mental stress test and assessed with echocardiography and Holter monitor [11]. The mental stress was recall of an upsetting situation and having to perform mental arithmetic, but the study had no control intervention. The primary endpoint was echocardiogram-derived myocardial performance index. There was no difference between their patient groups in any of the responses. As in our study, stress was found to increase SDNN, a marker of cardiac sympathetic activity.

Our results do not exclude a transient alteration in QT response as being a factor in a ‘perfect storm’ model for the causation of stress cardiomyopathy. In a perfect storm model, the hypothesis is that for a person to develop the condition, a number of genetic and environmental factors have to come together at a certain time and in certain manner. In stress cardiomyopathy, the most obvious factors are being female, being post-menopausal, and being subjected to sudden deeply personal emotional stress. On top of this, it could be hypothesised that the stressor might be more likely to trigger the condition if it occurs at a time of vulnerability, and the autonomic nervous system could be a factor. Unfortunately, we were not able to identify QT response as a marker for vulnerability.

In conclusion, QTc response in women with a history of stress cardiomyopathy does not differ from controls. The relevance of QT prolongation and sensitivity in the autonomic response to the pathogenesis of stress cardiomyopathy remains uncertain.

## Supporting information

S1 Protocol 24 hour Holter ECG monitoring to measure QT interval and autonomic function in patients with a history of takotsubo syndrome. (DOCX)

S1 File Hyperventilation and diaphragmatic breathing instructions to trial participants. (DOCX)

S1 Checklist A completed CONSORT checklist of information to include when reporting a clinical trial. (DOCX)
