# Supplementary material for: A randomised cross-over trial of QT response to hyperventilation-induced anxiety and diaphragmatic breathing in patients with stress cardiomyopathy and in control patients
Source: PLoS One. 2022 Mar 23;17(3):e0265607. doi: 10.1371/journal.pone.0265607 (PMC8942223; doi:10.1371/journal.pone.0265607)
Supplement: S1 Protocol — (DOCX) [file pone.0265607.s001.docx]

Protocol

24 hour holter ECG monitoring to measure QT interval and autonomic function in patients with a history of takotsubo syndrome

Dr Paul Bridgman and Dr Cameron Lacey

Departments of Cardiology and Psychological Medicine 2018

Background

There is widespread interest in the interaction between the mind and the body, and it is well accepted that psychological factors influence physical illnesses. The purest example of psychological stress causing a potentially fatal disease is takotsubo syndrome, also known as broken heart syndrome. In this syndrome the psychological stress that triggers the condition is typically acute and severe (and therefore readily identifiable) and the symptoms can develop within moments. Globally the incidence of takotsubo syndrome is increasing and in Christchurch in particular there is major interest in the syndrome. Three case clusters were precipitated by large earthquakes in Canterbury in 2010, 2011, and 2016. Community interest supports a significant program of research into the condition at Christchurch Hospital. This project builds on previous successful projects undertaken over the last 8 years.

Rationale

Experts agree that we still do not understand the mind-body interaction that triggers takotsubo syndrome. International studies have found that in normal people emotional arousal such as anxiety can influence the standard electrocardiogram, in particular the QT interval. Prolongation of the QT interval is a major feature of takotsubo syndrome. In this study we directly look at the mind-body interaction in patients with a history of takotsubo syndrome and compare them to patients who have had a previous hospitalisation with an acute coronary syndrome.

Note that internationally and in Christchurch over 95% of patients with takotsubo cardiomyopathy are female. Therefore only female patients will be included in this study

Major hypothesis

Study design

Outpatients will wear a holter monitor for 24 hours. This will allow for recording of their heart rhythm for a day of their usual activities. Monitoring will be performed on two occasions, with more than one week between each. On each day after the holter is fitted the patient will perform a 3 minute breathing exercise. Using a randomised cross-over design on one occasion this will be voluntary hyperventilation and on the other it will be controlled breathing.

Setting

Christchurch Hospital Cardiology Department.

Participants

Twenty four women with a past history of admission to hospital with either takotsubo syndrome or a non –ST elevation acute coronary syndrome (NSTEACS). There will be twelve in each group.

Takotsubo syndrome will be defined according to modified-Mayo criteria. That is a hospital presentation with a troponin elevation, a recognised regional wall motion pattern that fully resolves and absence of culprit coronary artery disease on coronary angiography.

Non-ST elevation acute coronary syndrome is defined as a hospital presentation with either unstable angina or non-ST elevation myocardial infarction.

Exclusion criteria: impaired left ventricular function (LVEF <50%)

current therapy with beta blockade

history of panic disorder

atrial fibrillation

left bundle branch block

The takotsubo syndrome cases will come from women who have previously consented to be a part of a case registry at Christchurch Hospital. The control subjects who have had an NSTEACS will be patients that are routinely being seen as part of their care in outpatient clinic or on the wards at Christchurch Hospital. They will be asked at the end of the consultation if they would like to look at being a control participant.

Interventions

The only interventions in this study will be the breathing exercises. These will be three minutes of voluntary hyperventilation and three minutes of controlled breathing. We will use a cross-over design with one of these exercises being performed at the start of each monitoring period. Breathing exercises will be performed with the participant seated.

During hyperventilation the participant is asked to consciously breathe deeper and faster for three minutes. Hyperventilation will be performed in a supervised setting. It is an autonomic nervous system stimulus that does not involve physical activity, the patient is seated. We are using it in this study in this manner as we will be able to accurately know the timing and can therefore correlate any autonomic changes seen on the ECG recording. Voluntary hyperventilation can cause a feeling of mild anxiety. The mild anxiety that may result is at a level similar to what may be encountered in normal everyday life. There is no specific risk from this. The supervisor will discontinue the hyperventilation if this develops. Hyperventilation may also cause a dry mouth as a direct effect of increased air flow. The supervisor will discontinue the exercise if the participant notes a dry mouth. Drinking water will also be available.

Controlled diaphragmatic breathing will serve as the control. It should not result in autonomic nervous system activation and from the patient perspective will be a similar exercise to the hyperventilation. Instructions to the patient are as follows:

- Take a long, slow breath in through your nose, first filling your lower lungs, then your upper lungs.
- Hold your breath to the count of "three."
- Exhale slowly through pursed lips, while you relax the muscles in your face, jaw, shoulders, and stomach.

There are no specific risks associated with three minutes of controlled diaphragmatic breathing.

Observations

Twenty four hour holter monitoring. This is a non-invasive test in which patients have ECG electrodes placed and carry the monitoring device for a day. Continuous ECG tracing is then downloaded for off-line analysis. There are no specific risks to holter monitoring.

PANAS Questionnaire. During the Holter monitoring day with the hyperventilation exercise the patients will be telephoned at 4 random times with the exact time documented by the investigator. At each of the phone calls the participant will be asked the questions of the PANAS questionnaire. This comprises 20 questions with the respondent giving a rating for each current emotion on a 1 to 5 Likert scale. The questionnaire is attached. It will take less than two minutes to complete on each occasion. It will provide a measure of emotional state to be correlated with the observed ECG changes in off-line analysis.

Primary and secondary outcome measures

Primary: Acute QT interval change with breathing exercises.

Secondary: QT interval change in the 24 hours of usual activity.

Heart rate variability (also available from the holter monitor as an index of autonomic function).

**PANAS questionnaire**

Use this PANAS questionnaire template to fill in the PANAS Scale. The PANAS scale lists different feelings and emotions on which you can link a score to, based on how you are feeling. At the bottom you can find scoring instructions.

**Time instructions**

There are different time instructions possible when using the PANAS Scale. In this test the response is for how you are feeling at the moment.

- Moment (you feel this way right now)

**Scale & Scorecard**

| 1 Very slightly or not at all | 2  A little | 3 Moderately | 4 Quite a bit | 5 Extremely |
| --- | --- | --- | --- | --- |

| **#** | **Score** | **Feelings/emotions** |
| --- | --- | --- |
| 1 |  | Interested |
| 2 |  | Guilty |
| 3 |  | Excited |
| 4 |  | Upset |
| 5 |  | Strong |
| 6 |  | Distressed |
| 7 |  | Scared |
| 8 |  | Hostile |
| 9 |  | Enthusiastic |
| 10 |  | Proud |

| **#** | **Score** | **Feelings/emotions** |
| --- | --- | --- |
| 11 |  | Irritable |
| 12 |  | Alert |
| 13 |  | Ashamed |
| 14 |  | Inspired |
| 15 |  | Nervous |
| 16 |  | Determined |
| 17 |  | Attentive |
| 18 |  | Jittery |
| 19 |  | Active |
| 20 |  | Afraid |

**Scoring instructions**

**Positive Affect Score**Add the scores on items 1, 3, 5, 9, 10, 12, 14, 16, 17 & 19. Scores can range between 10 – 50. Higher scores represent higher levels of positive affect. Mean scores: momentary = 29.7 and weekly = 33.3.

**Negative Affect Score**Add the scores on items 2, 4, 6, 7, 8, 11, 13, 15, 18 & 20. Scores can range between 10 – 50. Higher scores represent higher levels of negative affect. Mean scores: momentary = 14.8 and weekly = 17.4.
