# Supplementary material for: A randomised cross-over trial of QT response to hyperventilation-induced anxiety and diaphragmatic breathing in patients with stress cardiomyopathy and in control patients
Source: PLoS One. 2022 Mar 23;17(3):e0265607. doi: 10.1371/journal.pone.0265607 (PMC8942223; doi:10.1371/journal.pone.0265607)
Supplement: S1 File — (DOCX) [file pone.0265607.s002.docx]

# Hyperventilation and diaphragmatic breathing instructions to patient

In both interventions, the participant was given the instructions as reproduced below, and reminded of the instructions when it appeared they were deviating from them.

Hyperventilation:

When the timer starts, I want you to take big breaths in and out through your nose or mouth. Make each breath as hard and fast as you can.

Diaphragmatic breathing:

When the timer starts, I want you to take a long, slow breath in through your nose, first filling your lower lungs, then your upper lungs. Hold your breath to the count of "three", and then exhale slowly through pursed lips, while you relax the muscles in your face, jaw, shoulders, and stomach.
